# A hybrid optimization with ensemble learning to ensure VANET network stability based on performance analysis

**DOI:** 10.1038/s41598-022-14255-1

**Published:** 2022-06-18

**Authors:** Gagan Preet Kour Marwah, Anuj Jain

**Affiliations:** School of Electronics and Electrical Engineering, Lovely Professional University, Phagwara, Punjab India

**Keywords:** Electrical and electronic engineering, Engineering

## Abstract

High vehicle mobility, changing vehicle density and dynamic inter-vehicle spacing are all important issues in the VANET environment. As a result, a better routing protocol improves VANET overall performance by permitting frequent service availability. Therefore, an ensemble-based machine-learning technique is used to forecast VANET mobility. Effective routing based on a hybrid metaheuristic algorithm combined with Ensemble Learning yields significantly improved results. Based on information collected from the Road Side Unit (RSU) or the Base Station, a hybrid metaheuristic (Seagull optimization and Artificial Fish Swarm Optimization) method is used to estimate (BS). The suggested approach incorporates an ensemble machine learning and hybrid metaheuristic method to reduce the latency. The current model's execution is calculated using a variety of Machine Learning techniques, including SVM, Nave Bayes, ANN, and Decision Tree. As a result, the performance of machine learning algorithms may be studied and used to achieve the best results. Comparative analysis between the proposed method (HFSA-VANET) and (CRSM-VANET was done on different performance parameters like throughput, delay, drop, network lifetime, and energy consumption to assess system performance on two factors Speed and Nodes. The HFSA-VANET method shows an overall drop in the delay of 33% and a decrease in the energy consumption of 81% and an increase of 8% in the throughput as compared with the CRSM-VANET method at 80 node. The proposed method that is HFSA-VANET has been implemented in the MATLAB and NS2 environment.

## Introduction

Connected and Automated Vehicles (CAVs) have been the subject of extreme research over the last 10 years and look to provide a solution to various consideration with Intelligent Transportation Systems (ITSs)^1^. Various researchers, businesses, and governments throughout the world are devoting significant time and resources to the development of ITS based on wireless communication networks^2^. Wireless networks connect automobiles to ease information exchange between Automobiles, improving the security and ease of drivers and passengers. Broadcasting is a common approach in the VANET^3^. Broadcast might be used for exchanging crisis, traffic, weather, and transportation data among vehicles and sending commercials and notifications^4^. Broadcast routing in VANETs differs significantly from routing in mobile ad hoc networks (MANET) due to a variety of characteristics such as extremely dynamic topology, frequent disconnections, appropriate energy, and so on. As a result, designing an efficient routing system for VANET is critical. Because VANET automobiles drive at such high speeds, dynamic changes in topology occur often, offering a number of challenges in routing research^5^. In a VANET, for example, vehicle travel is normally limited to bidirectional movements restricted to highways and streets. The activity of automobiles in VANET is limited by the roads and streets, they tend to be predictable^6^. As a result, each vehicle may keep track of its previous movements as well as its current location. Vehicles may forecast the future movement condition of their neighbors and themselves by doing so.

Furthermore, in position-based routing, it is believed that the communications partner's positional coordinates are already known^7^. As a result, position-based routing is widely considered one of the most favourable VANET technologies. Broadcast techniques for ad hoc networks have a large body of literature^8^. Because of the restricted amount of space available, they concentrate efforts on a small number of solutions that better meet the requirements of VANETs^9^. Flooding, in which every automobile re-showing messages to all of its neighbors beside the one it received from, is the effortless attitude to make a broadcast service. Flooding ensures that the message reaches all nodes in the network at some point and achieves good performance for a short supply of nodes^10^. Also, it is easily operated. However, as the number of nodes in the network grows, performance dramatically suffers. To have a higher tolerance for node movement, Position-based Schemes are need nodes to be aware of that position^11^. Therefore, the furthest node is always chosen first, to maximize the covered area by each transmission rebroadcast nodes are used. Such as, The UMB protocol (Urban Multi-Hop Broadcast) is designed for broadcast propagation in automobile networks^12^. In UMB, every hop, the source nodes attempt to rebroadcast the message to the farthest node in the broadcast direction, requiring at least two-way handshakes^13^. Under large packet loads and high automobile traffic densities, the UMB protocol has a greater success rate than the 802.11-distance and 802.11-random protocols^14^. The possible relay nodes, on the other hand, wait for the longest before retransmission, depending on the congestion resolution mechanism. This method may cause a significant delay, particularly in high-mobility circumstances^15^. Vector-based Tracking Detection (V-TRADE) and History-enhanced V-TRADE, are position-based broadcast protocols are also suggested (HV-TRADE). Their primary opinion is comparable to the Global Positioning System-based unicast routing technology (GPS).

## Literature review

This section mainly covers the literature related to VANET based various research articles. In 2021, Nadarajan et al.^16^ have utilized the next generation VANET, machine intelligence was used to create a QoS aware and safe routing algorithm. Adaptive Routing with Prediction based on Stochastic Chaos (SCARP) advocates a stochastic-based node discovery routing concept because it can make effective traffic flow forecasts utilizing powerful deep learning networks. The method was decreased connectivity loss, lowered latency, and to create an efficient and secure data transmission system among automobile nodes, chaotic authentication is granted.

In 2020, Boukerche et al.^17^ introduced that the hybrid machine learning-based model was used to copy and analyze the task of a rare automobile traffic flow forecast system. They focus on strengthening the characteristics of the prediction model that is used for putting the model into practice. They offer a new hybrid deep learning model based on the Graph Convolutional Network (GCN) and the Gated Recurrent Unit (GRU) deep aggregation structure. The online prediction technique is based on refinement learning for the online prediction challenge. An efficient parallel training technique is applied to ITS to increase the model's accuracy and efficiency while making use of the vehicular cloud structure.

In 2021, Hossain et al.^18^ had presented the cognitive radio vehicular network via machine learning-based cooperative spectrum sensing in dynamic segmentation. According, to the probability value roads are separated into equal parts and then Partitioned. Particular cars or other users provide local sensing results by the combination of fuzzy and Nave Bayes methods that are utilized by a hybrid machine learning algorithm to choose the best spectrum sensing (SS) methodology. The segment spectrum agent (SSA) used the tri-agent reinforcement learning (TA-RL) technique to make the global choice in cooperative SS.

In 2019, Tang et al.^19^ had presented Machine-learning techniques to a potential knowledge and secure automobile network in the 6G era. The automobile network becomes diverse, active, and mass-scale, making it impossible to satisfy the tough measures of the next-generation (6G) network, such as ultralow latency, high reliability, high safety, and massive connections. For improving the ability and adaptability of both automobiles and wireless communication, Machine learning (ML) has developed as a potent artificial intelligence (AI) technology. To enable AI in a future 6G vehicular network, development of intelligent radio (IR) network, and self-learning with proactive exploration and some various machine learning techniques applied to communication, networking, and security parts in vehicular networks.

In 2019, Najm et al.^20^ have examined the best way to improve obstruction control in 5G IoT (Internet of Things) networks' wireless sensors. The model was used to discover the best parametric setting in a 5G scenario using a training dataset. The record was applied to prepare the machine learning model, which allowed for the prediction of optimal alternatives to improve the obstruction control approach's performance DT (decision tree) algorithms provide graphs that any user may use to understand how the prediction method works.

In 2021, Kaushik et al.^21^ have explored Wireless Ad Hoc Networks, a Futuristic Analysis of Machine Learning-Based Routing Protocols. The effects of machine learning on various wireless ad-hoc networks are investigated, as well as how performance characteristics shift as a result. All simulators that are used and assessed by different protocols used in MANET and VANET are studied using a methodical way. Many additional characteristics must be investigated and simulations utilized in Ad-hoc networks to enable users to assure optimization so that reduced the odds of data failure and increase throughput after picking the optimal machine learning model.

In 2019, Ye and Guan et al.^22^ had presented routing protocol by the way of MPBRP-mobility prediction. For vicinity recognition, packet transmission, and path recovery in VANETs a novel Mobility Prediction Based Routing Protocol (MPBRP) was designed using driver intent received from positioning systems. To discover neighbors and transmit packets, a predictive forwarding approach and a recovery technique are combined. To choose the surrounding nodes and identify the transmission line, consider the driver's aim in a designated time by using the expected location and angles. The recommended routing protocol exceeds current protocols in terms of packet delivery ratio and end-to-end latency, which increased entire execution.

In 2019, Abdel-Halim et al.^23^ have presented the VANETs, an effective clustering strategy for connected and autonomous cars was based on mobility prediction. The MPECS (Mobility Prediction-based Efficient Clustering Scheme) system was radical. The MPECS was to use a Voronoi diagram to partition the whole region into discrete sections, allowing each vehicle to forecast its lifetime and cost of being the cluster leader. Also, MPECS was compared to four current clustering techniques for VANETs and its performance was evaluated via simulation. The results of the tests reveal a high degree of consistency between simulation and analytical findings, demonstrating that MPECS may greatly increase clustering architectural stability with low overhead.

In 2020, Rehman et al.^24^ have investigated the VANETs influence of mobility speed on overall communication. The influence of vehicle speed disparities on the performance of two frequently used VANET messaging systems, the farthest distance and link quality-based techniques were investigated. The explored solutions use a sender-oriented relay selection mechanism to make the best use of channel bandwidth. The connection quality strategy surpasses the longer distance technique in both speed difference scenarios when it comes to message reach ability. As a result, the link quality was more resilient to the negative impacts of huge disparities in mobility speed than the farther distance plan.

In 2019, Abdellah et al.^25^ have utilized the robust VANET performance estimation. The training data mean square error (MSE) is used to minimize a conventional back propagation learning algorithm that is used by Neural networks. Using robust neural network learning based on the robust M-estimators presentation function rather than the usual MSE performance function, the VANET performance in terms of packet loss ratio and output. In terms of RMSE and training time, MSE performance functions exceed by robust M-estimators performance functions.

In 2021, Hui Liu et al.^26^ presented a new hybrid model where oil temperature forecasting technology can realize real-time detection of the gearbox status of wind turbines. The main modeling process of the presented method consists of three main steps. In step I, the proposed secondary decomposition method is utilized to preprocess the raw oil temperature data. In step II, the feature selection algorithm based on reinforcement learning selects the features of each sub-series. In step III, the simple recurrent unit network establishes forecasting models for each sub-series after feature selection and obtains the final forecasting results. After analyzing the forecasting results of multiple experiments, it can be concluded that the presented hybrid model can obtain satisfying forecasting results. It improves the performance of traditional neural networks by over 90 percent.

In 2021, Shuqin Dong et al.^27^ proposed traffic speed forecasting technology that can provide technical support to solve traffic congestion and ensure travel safety. In this paper, to build a traffic speed forecasting model with higher capacity, a new ensemble reinforcement learning gated recursive network model, which was composed of the LSTM network, the GRU network, and reinforcement learning-based ensemble learning was proposed. The model contained the LSTM network and the GRU network as predictors for in-depth mining of the characteristics of traffic speed data and used reinforcement learning to integrate the results of the two predictors, combining the advantages of multiple predictors to achieve stable and accurate forecasting results of traffic speed.

In 2021, Yan et al.^28^, proposed a novel axle temperature forecasting model by integrating the CEEMD method, the BILSTM neural network, and the PSOGSA optimization algorithm. In the proposed framework, the CEEMD was used to preprocess the raw irregular data into a set of sub-layers, which can facilitate the prediction of the next step. The BILSTM is applied for the prediction for each sub-layer. The PSOGSA algorithm would continue optimizing the initial value of forecasting results from each sub-layer and combine them for the final data. To study the forecasting capability of the proposed CEEMD-BILSTM-PSOGSA model, other benchmark predictors and hybrid models are listed and observed in the comparative research. A sensitive analysis of the hybrid model was also conducted to test its robustness and stability. The results proved that the proposed model can obtain the best prediction results with fewer errors between the comparative models and effectively represent the changing trend in axle temperature.

## Proposed methodology

### Background model of the Seagull Optimization algorithm

Two key processes are contained by The Seagull Optimization Algorithm (SOA): migration and attacking, which aid in the effective selection of the cluster leader. Initially, the Migration phase simulates gulls migrating from one site to another. The three core requirements of the Seagull are Collision avoidance, movement in the direction of optimal neighbours, and staying near to the best search agent.

#### Collision avoidance

Using additional variable A*, the collision between neighbour search agents is decreased in this phase. The "A*" is used to determine the new search agent location which is represented by,

1 $${P}_{s}^{*}={A}^{*}\times {P}_{S}^{*}\left(\mathrm{x}\right).$$

Although, ${P}_{s}^{*}$ presents the position of a search agent that avoids colliding with other search agents, ${P}_{S}^{*}$(x) indicates where the search agent is in the current iteration or the position, and ${A}^{*}$ depicts how search agents move around in a given search space.

2 $${A}^{*}={f}_{A}-\left({X}^{^{\prime}}\times \left(\frac{{f}_{A}}{{MAX}_{iter}^{*}}\right)\right).$$

To control the frequency of the variable “${A}^{*}$”, “${f}_{A}$” is used where f_A_ is set to be 2 then it will be reduced linearly to "zero". The search agent indicates in the best vicinity when the collision is prevented.

#### A movement towards best neighbors’ direction

3 $${P}_{SA}={B}^{*}\times \left({P}_{bs}^{*}\left(x\right)-{P}_{S}^{*}\left(\mathrm{x}\right)\right).$$

Although, ${P}_{SA}$ presents the site of Search agent ${P}_{s}^{*}$ to the best search agent position ${P}_{bs}^{*}$. The variable "${B}^{*}$" is used at random to manage the balance between exploration and exploitation in this case. The following equation shows the mathematical derivation of ${B}^{*}$.

4 $${B}^{*}=2\times {A}^{2*}\times RD.$$

Although, the random value [0,1] are represented by RD. Atlast, using the following equation, the position of the search agent is updated based on the optimal search agent.

#### Approaching the best position

5 $${D}_{S}^{*}=\left|{P}_{s}^{*}+{P}_{SA}\right|.$$

Although, ${D}_{S}^{*}$ represents the distance between the search agent and the best search agent.

This method is based on a review of previous search methods. The Spiraling move is used to assault prey while in the air. Three planes ${X}^{^{\prime}}$, ${Y}^{^{\prime}}$, and ${Z}^{^{\prime}}$ are important in this procedure. The following is a description of each plane.

6 $${X}^{^{\prime}}={R}^{^{\prime}}\times Cos\, {K}^{^{\prime}},$$

7 $${Y}^{^{\prime}}={R}^{^{\prime}}\times Sin\, {K}^{^{\prime}},$$

8 $${Z}^{^{\prime}}={R}^{^{\prime}}\times {K}^{^{\prime}},$$

9 $${R}^{^{\prime}}={U}^{^{\prime}}\times {e}^{{K}^{^{\prime}}{V}^{^{\prime}}},$$

where, ${R}^{^{\prime}}$ signifies that the related component is taken into account for each turn radius. ${U}^{^{\prime}}$ and ${V}^{^{\prime}},$ the spiral is formed by multiple turns in the search space. The spiral form is defined by these consonants. ${K}^{^{\prime}}$ means a Random number that range is [0≤ ${K}^{^{\prime}}$ ≤ 2π]. e is thebase of the actual logarithm.

The Eqs. (8) and (9) are utilized for updating the position of the search agent.

10 $${P}_{S}^{*}\left(\mathrm{x}\right)=\left({D}_{S}^{*}\times {X}^{^{\prime}} \times {Y}^{^{\prime}} \times {Z}^{^{\prime}}\right)+ {P}_{bs}^{*}\left(x\right),$$

where, ${P}_{s}^{*}$ represents the best result are saved and updated the other search agents.

### Improved artificial fish swarm algorithm

The artificial fish swarm algorithm (AFSA) is a novel design methodology that creates a swarm of synthetic fish that mimics the properties of real fish in water. Through simple low-level conduct and local connection of individuals, the algorithm indicates the group's high artificial intelligence conduct on a macro level. In the algorithm optimization process, it is presented as local optimization of users and optimal global approximation of swarms. Standard AFSA is an inhabitant’s random methodology which usually begins with a set of the randomly generated initial inhabitants and then converges to determine the best resolution.

#### Initialization

Consider that the fish population a number of nodes or vehicles that is represented as N at the beginning, and that the issue at stake is a D-dimensional problem. One fish's condition is Computed by the fish swarm's state vector that can be described as:

11 $${X}_{i}=\left({x}_{1},{x}_{2},\dots ,{x}_{D}\right).$$

Here, the fish swarm’s state vector is represented as the nodes or the vehicle’s state. Then The food satisfaction [QoS (bandwidth, distance)] is described by Yi = f(Xi). The Euclidean distance, which describes any two fish's interaction, is defined as

12 $${D}_{S}^{*}=\Vert {X}_{i}-{X}_{j}\Vert .$$

For $i,j=\mathrm{1,2},\dots ,N,{X}_{i}$ and ${X}_{j}$ are the various states of the fish or nodes (Vehicles). The visual field of the fish, the step size, and the degree of crowding, described by Visual, Step, and Crowded factor $\delta$, respectively, are the parameters that influence the algorithm's performance. The exploration process of sensor nodes in sensor networks regarding greater network coverage levels is akin to the approach activity of artificial fish towards great meal satisfaction. All artificial fish just use characteristics of Random, Preying, Swarming, and Following to find an optimal feeding source.

##### Vehicle position based on improved unspecified conduct

Random conduct is described as the Action of a fish moving randomly in their visual field. If the fish's current state $i$ is ${P}_{s}^{*}$ (Current State obtained through Seagull optimization), it will choose a state at random out of its visual field. The following is the process for moving:

13 $${{P}_{s}^{*}}_{/next}={P}_{s}^{*}+visual+Rand().$$

Here, visual field of the fish is denoted by Visual, and random number is denoted by Rand() ranges from 0 and 1. The obtained current vehicle position ${P}_{s}^{*}$ from Seagull Optimization is to improve with AFSO-VANET.

##### Preying conduct

Preying activity is termed as the action of a fish moving randomly in the water, hunting for food, and moving towards a place with high food satisfaction. If Xi denotes the vehicle’s current state, and it selects a new state ${X}_{j}$ at random within its visual field, such that:

14 $${X}_{j}={P}_{s}^{*}+visual\times {Rand\left(\right)}.$$

Consider if $f({P}_{s}^{*})$ and $f({X}_{j})$ indicate the state ${P}_{s}^{*}$ and state Xj food pleasure, accordingly.

*Case 1* $f({P}_{s}^{*})<f({X}_{j})$ A fish performs a single step forward in the next direction at its maximum, and its current state ${P}_{s}^{*}$ next can be expressed as:

15 $$X_{{i/next}} = P_{s}^{*} + \frac{{P_{s}^{*} - X_{j} }}{{\left[\kern-0.15em\left[ {X_{j} - P_{s}^{*} } \right]\kern-0.15em\right]}} \times step \times Rand\left( {} \right),$$

where ‖⋅‖ is the Euclidean distance between the artificial fish i and the artificial fish j.

*Case 2* $f({P}_{s}^{*})\ge f({X}_{j})$ The artificial fish reselects other states randomly. If the fish cannot meet the needs in a particular time, it moves one step randomly as (15).

##### Collision avoidance in packed tendency to traffic management

The VANET environment is incorporated with an abundance of vehicles that cause huge traffic and lead to an accident. Therefore, the intelligence of the HFSA-VANET to manage the traffic using the idea of AFSO is improved by SOA strategy. In AFSO based VANET, Swarming tendency describes the tendency of artificial fish to assemble in sets and draw near to the middle of their neighbors’ nodes (vehicles). Let Xi reflect the fake fish's present condition, which is replaced with the updated best position of SEO “${P}_{s}^{*}$”, n_f_ refers to the number of neighbor nodes inside the visual field, and Xc indicates the fellows' center position state. The following formula was used to calculate the condition of the centre position:

16 $${X}_{c}=\left({\sum }_{j=1}^{{n}_{f}}\frac{{X}_{j}}{{n}_{f}}\right),{D}_{IJ}<visual.$$

Euclidean distance between the artificial fish i and the artificial fish j is denoted by ${D}_{ij}$.

The uncrowded food satisfaction of center position state X_c_ is f(X_c_). If f(X_c_)/n_f_ > δf(${P}_{s}^{*}$).Whereas δ is the crowded factor. If $f({X}_{c})\ge f({P}_{s}^{*})$ the fish moves one step ahead by the follows center position as follows:

17 $$P_{{s\,/next}}^{*} = P_{s}^{*} + \frac{{X_{c} - P_{s}^{*} }}{{\left[\kern-0.15em\left[ {X_{c} - P_{s}^{*} } \right]\kern-0.15em\right]}} \times step \times Rand\left( {} \right).$$

If this conduct is not profitable, the fish will indulge in preying behavior. The magnitude of swarms is successfully limited by the degree of crowdedness. The higher the food gratification, the bigger the fish swarming scale (the better Qos value).

With the inclusion of the Seagull Optimization with the Artificial Fish Swarm Optimization, the collision avoidance and movements of the vehicles can be managed properly.

##### Following conduct

The fake fish with the highest food fulfilment in the visual field migrate nearer to their vicinity as a result of their following conduct.

${P}_{s}^{*}$, denotes the current state of the artificial fish, n_f_ denotes number of its vicinity within the visual field. In case, X_j_ stands for the best fish (in the best food position) within the visual field of Xi. The food gratification of situation state X_j_ is f(X_j_). If f(X_j_)/nf > δf(${P}_{s}^{*}$) and f(X_j_) > f(${P}_{s}^{*}$), the fish moves one step forward X_j_

18 $$P_{{s\,/next}}^{*} = P_{s}^{*} + \frac{{B^{*} (P_{s}^{*} - X_{j} )}}{{B^{*} \left[\kern-0.15em\left[ {X_{j} - P_{s}^{*} } \right]\kern-0.15em\right]}} \times step \times Rand\left( {} \right).$$

**Figure 1:**
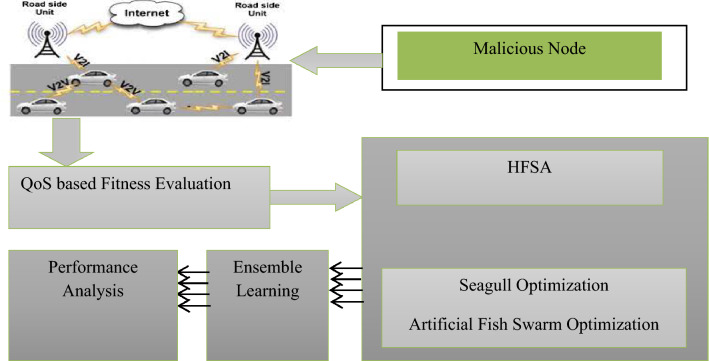
Overall architecture of the proposed model.

**Figure 2:**
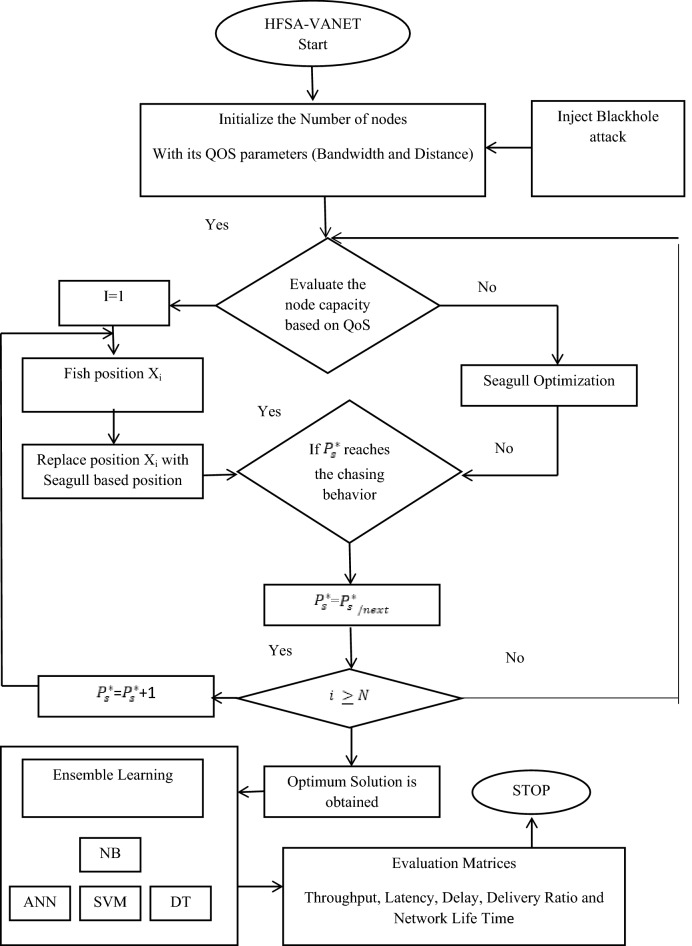
Flowchart for HFSA-VANET.

In Eq. (18), the variable ${B}^{*}$ is added with the following behaviour to improve its performance based on the behaviour of the seagull optimization algorithm. The algorithm is terminated when all of the iterations of the entire algorithm have indeed been finished and the ideal scenario is the state recorded on the bulletin board. Here, the Cluster Head selection based on Fitness value is highly significant. Then the process is repeated to the neighbor node in order to ensure that all the nodes are assigned with the best fitness value. On other hand, an Ensemble learning approach is to mitigate the blackhole attack in the network areas. This kind of attack degrades the performance of the network efficiency; therefore, an ensemble learning approach based on ANN, SVM, DT, and Navie Bayes algorithms is utilized to evaluate the performance. In this model, the obtained results through HFSA-VANET are evaluated with all the techniques in Ensemble Learning. Each produces different results separately, that will help to determine the best results by identifying and determining the attacks. The flowchart of the proposed model shows that how the proposed model obtained better results with HFSA-VANET with the ensemble approach. The Figs. 1 and 2 illustrate the overall architecture and flowchart respectively.

### Ethics approval

It is a simulation-based design and analysis. So, it does not affect any human being or animal.

## Results and discussion

### Section 1

Section 1 contains the results and discussion of the proposed and implemented methods for improving machine learning with a hybrid optimization strategy to predict mobility in VANET. The execution of the project (HFSA-VANET) is evaluated and compared to that of current method (CRSM-VANET). Delay, Energy consumption, Drop, Throughput, and Fairness index measured values are computed and compared to propose (HFSA-VANET) and existing (CRSM-VANET)^29^ methods. In addition, the implementation is done through NS2 stimulation and comparing the proposed algorithm with these two platforms, along with the windows 10 PRO computer, total RAM capacity of 10 GB, and processor utilized is Intel^®^ core (7M) i3-6100CPU @ 3.70 GHz processor. The performance metrics are examined in the next section.

#### Performance metrics

- Delay

Delays occur while a packet travels from its source to its destination.

19 $$delay= \frac{length}{bandwidth}.$$

- Drop

It is the number of packets lost as a result of a rogue node (DoS attack).

20 $$Drop=\frac{Send \;packet-Received \;packet}{Send \; packet}.$$

- Throughput

The throughput refers to the amount of packet data established across a destination, which corresponds to the overall value of packets created by the sender node within a certain time. The formula is as follows:

21 $$\mathrm{Throughput}\hspace{0.17em}=\hspace{0.17em}\mathrm{received \; data \; packet }\times 8/\mathrm{data \; packet \; transmission \; period}.$$

#### Results obtained through node

The performance metrics of the existing technique and the proposed method is compared in the table below.

The primary goal of the performance metrics is to assess the proposed model's ability to predict mobility in VANET. According to Table 1, when compared to and examined with existing methodology, the proposed method improves machine learning with a hybrid optimization strategy to predict mobility in VANET is more successful.

**Table 1:** Comparison with existing approach.

Node	Delay		Energy consumption		Drop		Throughput		Fairness Index (FI)	
	Proposed method (HFSA-VANET)	Existing method (CRSM-VANET)	Proposed method (HFSA-VANET)	Existing method (CRSM-VANET)	Proposed method (HFSA-VANET)	Existing method (CRSM-VANET)	Proposed method (HFSA-VANET)	Existing method (CRSM-VANET)	Proposed method (HFSA-VANET)	Existing method (CRSM-VANET)
20	0.09369	2.123612	99	99	0.897708	0.998142	31,341	30,689	7	8
40	9.752925	12.431245	47	58	0.472094	0.586371	30,573	29,382	3	4
60	10.902826	13.786562	36	50	0.376633	0.514273	28,423	26,749	2	4
80	15.287826	20.464329	11	31	0.116375	0.310394	18,197	16,739	1	2

The delay, energy consumption, drop, throughput and fairness index of the HFSA-VANET and the CRSM-VANET are compared below.

**Figure 3:**
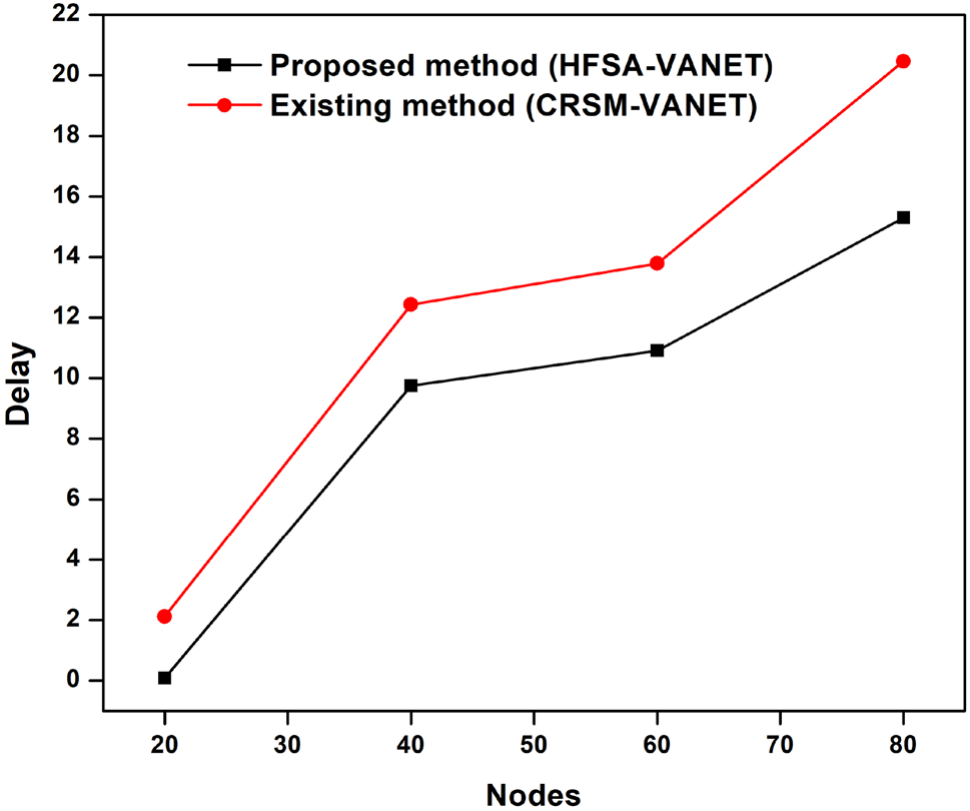
Delay plot for a proposed and existing method.

**Figure 4:**
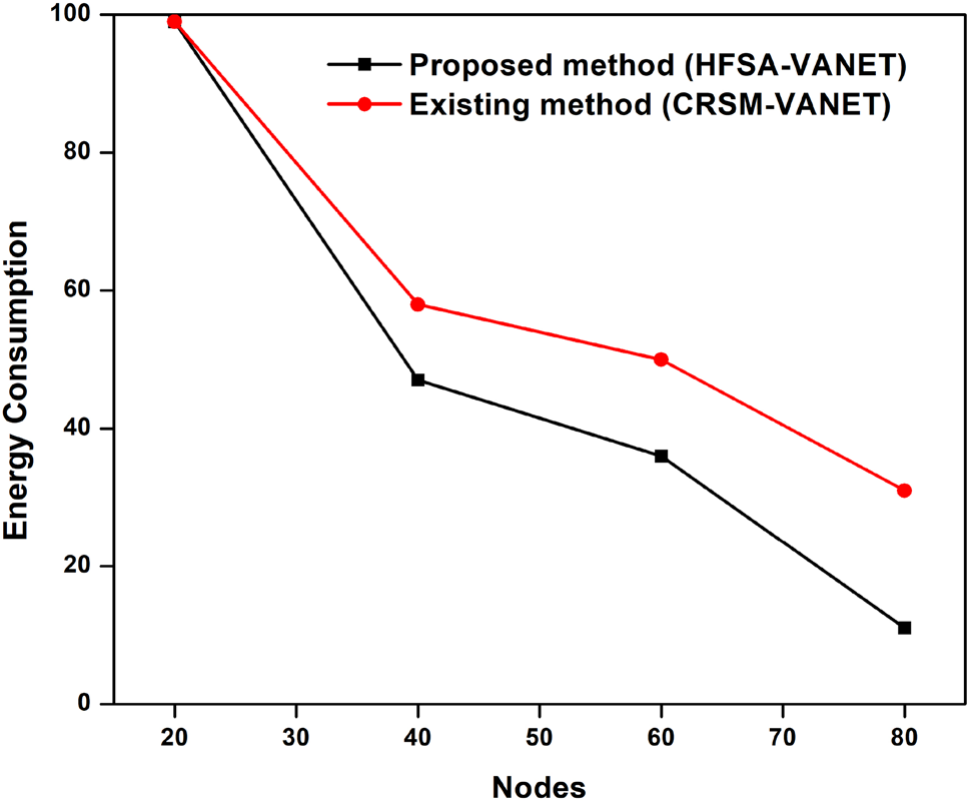
Energy consumption plot for a proposed and existing method.

**Figure 5:**
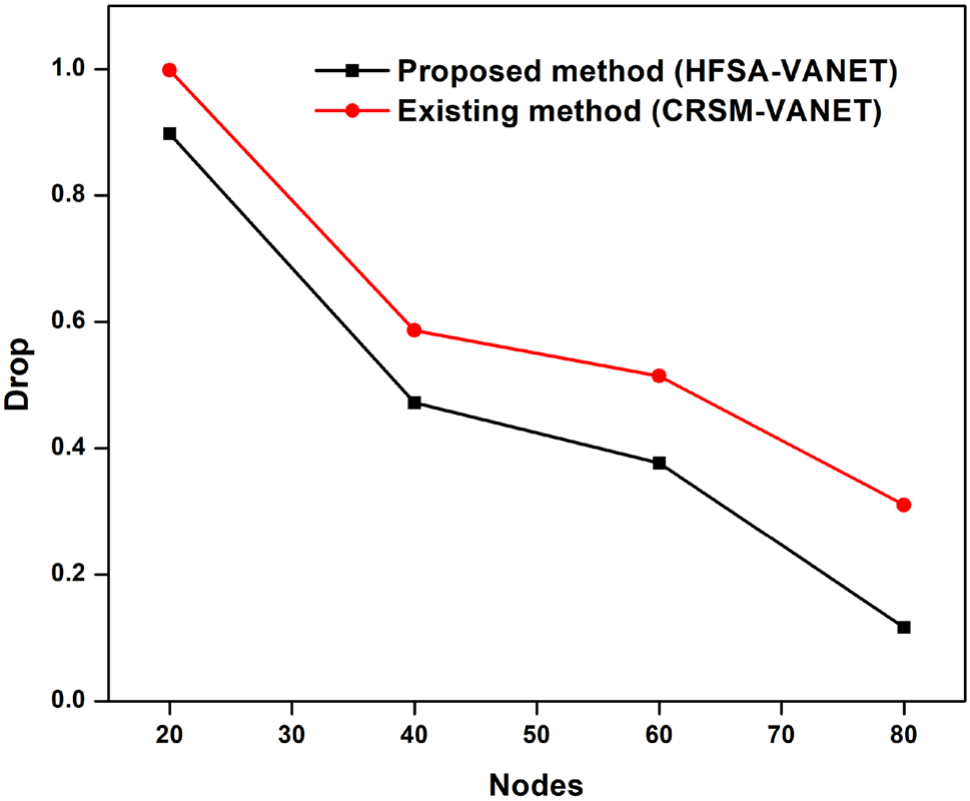
Drop plot for proposed and existing method.

**Figure 6:**
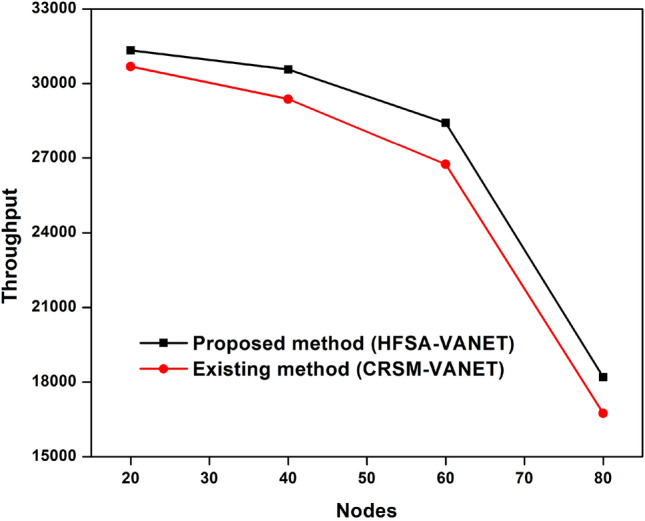
Throughput plot for proposed and existing method.

**Figure 7:**
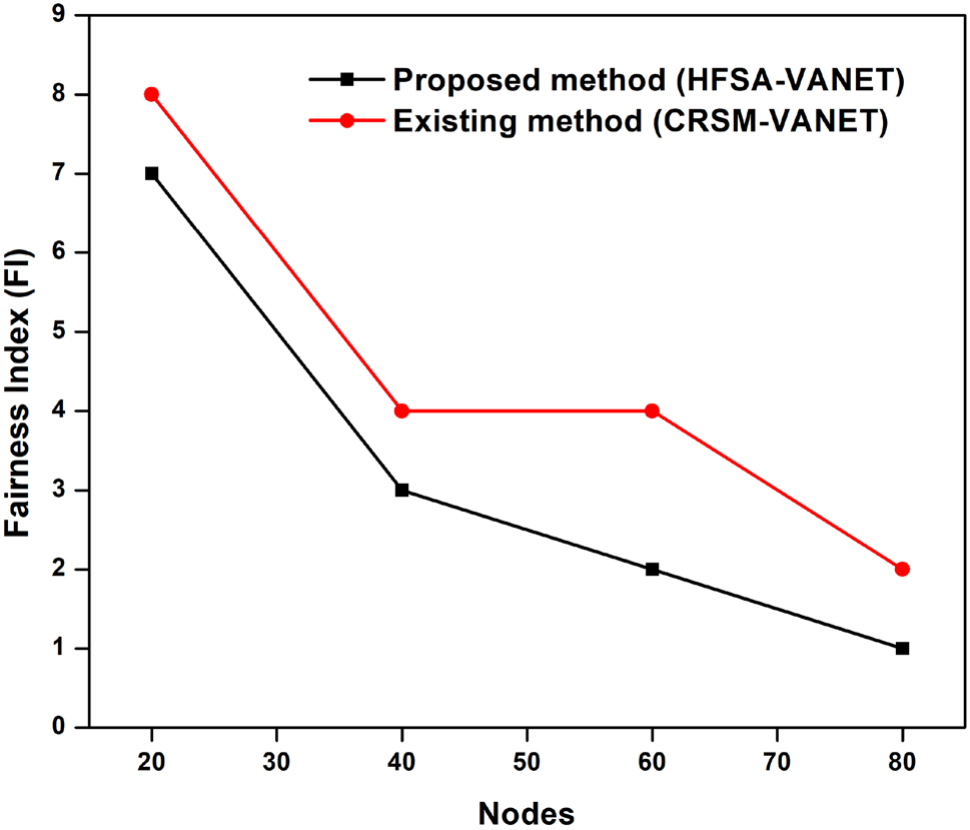
Fairness Index plot for the proposed and existing method.

Suggested technique achieves 99 J, 0.093690, 0.897708 for energy consumption, delay value, and drop value in node 20. Furthermore, the new technique achieves a Throughput of 31,341, which is higher than the prior approach. The proposed technique has a fairness score of 7.000000, whereas the current method has a value of 8.000000. For energy consumption, delay value, and drop value in node 60, the proposed approach achieves 47 J, 9.752925, 0.472094. In addition, the new method obtains a Throughput of 31,341, which is greater than the previous method. The suggested strategy has a fairness score of 3.000000, compared to 4.000000 for the present method. The proposed technique achieves 36 J, 10.902826, 0.376633 for energy consumption, delay value, and drop value in node 60. Furthermore, the suggested technique achieves a Throughput of 28,423 compared to 26,749 for the existing method. A fairness index value of 2.000000 for the proposed method vs 4.000000 for the existing method is achieved. For energy consumption, delay value, and drop value in node 80, the suggested approach achieves 11 J, 15.287826, 0.116375. Furthermore, as compared to the previous approach, the proposed strategy obtains a Throughput of 18,197. The proposed approach has a fairness index of 1.000000, whereas the present method has a fairness score of 2.000000. The Figs. 3, 4, 5, 6 and 7 are Delay, Energy Consumption, Drop, Throughput, Fairness Index are obtained through node, respectively.

#### Results obtained through speed

The speed of the proposed technique and the existing techniques are compared in terms of delay, energy consumption, drop, throughput, and fairness index. The measured values are demonstrated in the table below. Table 2 shows the speed values of both existing and proposed techniques.

**Table 2:** Comparison of the proposed method to existing method of speed.

Speed	Delay		Energy consumption		Drop		Throughput		Fairness Index (FI)	
	Proposed method (HFSA-VANET)	Existing method (CRSM-VANET)	Proposed method (HFSA-VANET)	Existing method (CRSM-VANET)	Proposed method (HFSA-VANET)	Existing method (CRSM-VANET)	Proposed method (HFSA-VANET)	Existing method (CRSM-VANET)	Proposed method (HFSA-VANET)	Existing method (CRSM-VANET)
20	1.873793	2.47224	1980	1980	19.95416	19.96283	150	90	6	6
40	390.117	497.249781	1880	2320	18.883762	23.45486	35	26	3	4
60	654.169557	827.19374	2220	3060	22.597974	30.85639	22	16	2	3
80	1223.026093	1637.146333	880	2480	9.309993	24.8315	8	6	0	2

**Figure 8:**
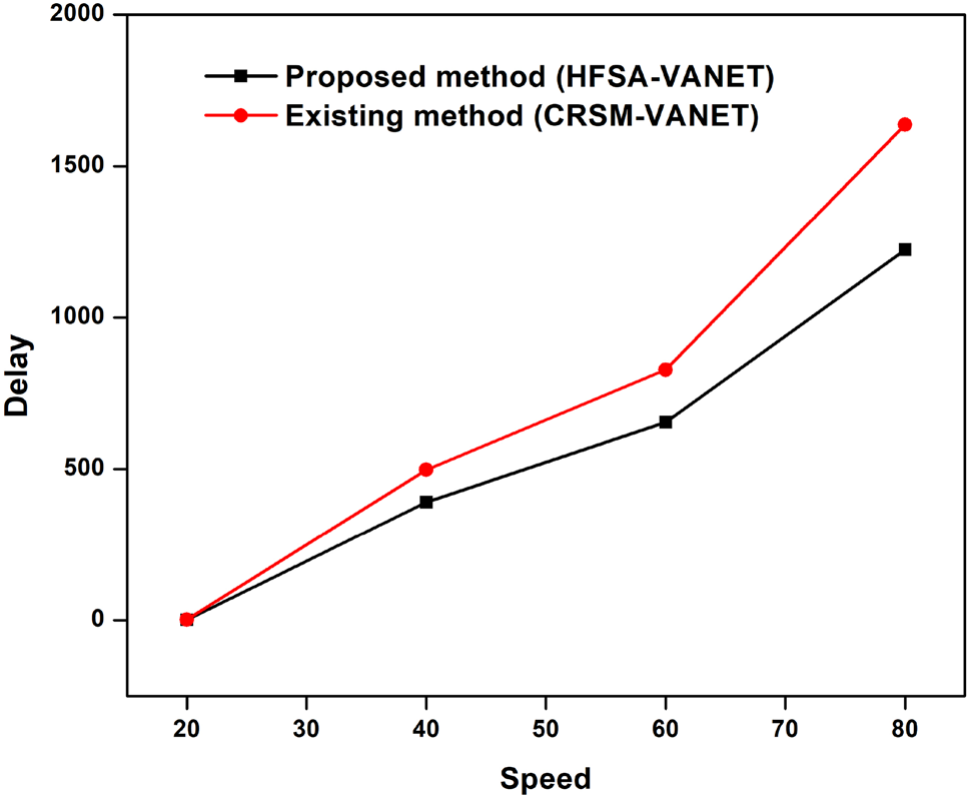
Speed vs delay plot for proposed and existing method.

**Figure 9:**
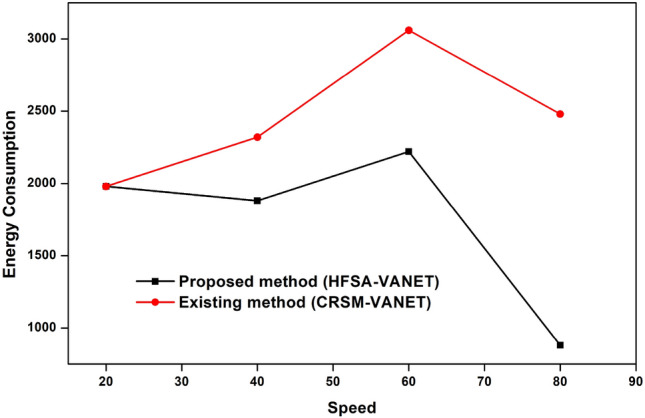
Speed vs energy consumption plot for a proposed and existing method.

**Figure 10:**
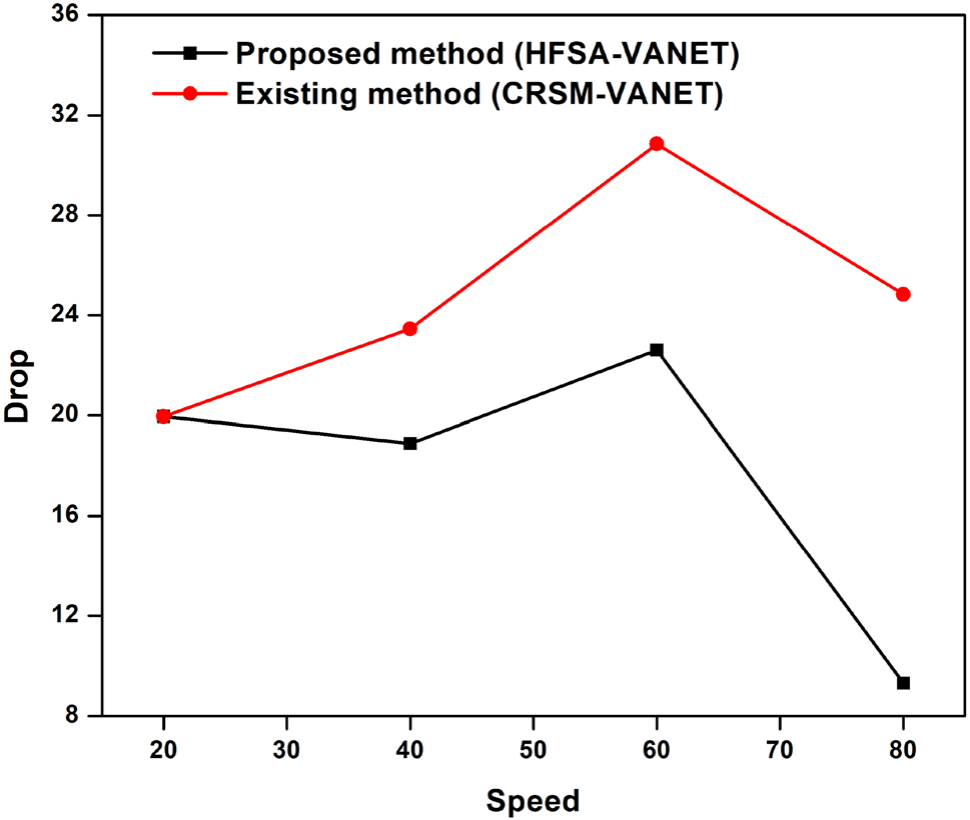
Speed vs drop plot for a proposed and existing method.

**Figure 11:**
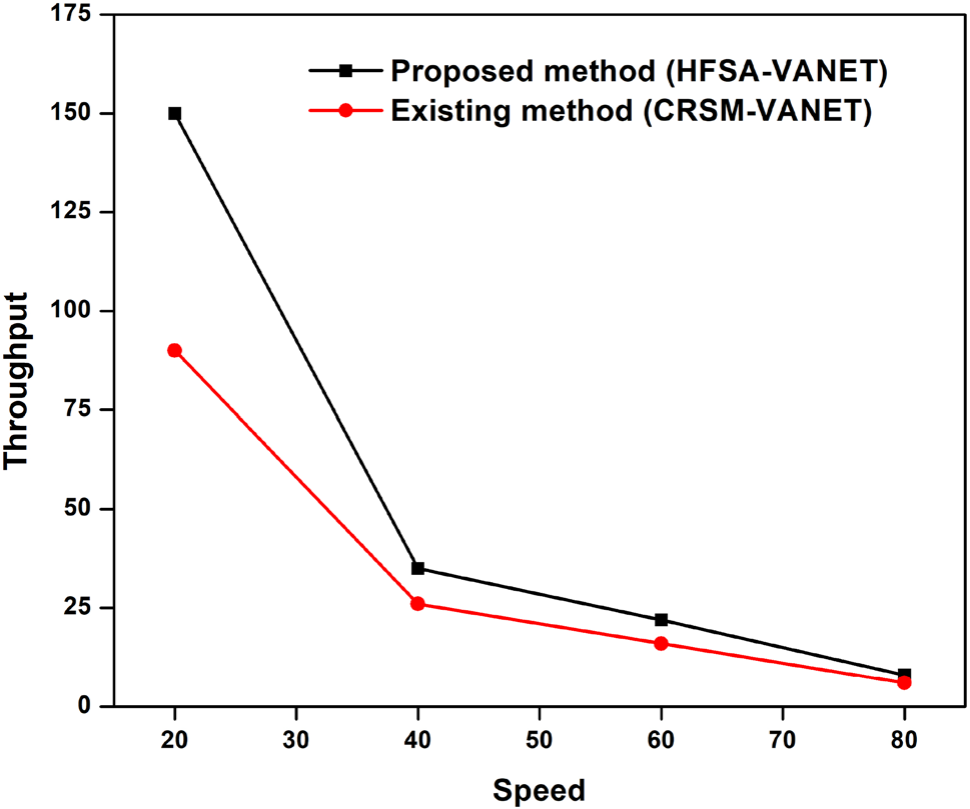
Speed vs throughput plot for a proposed and existing method.

**Figure 12:**
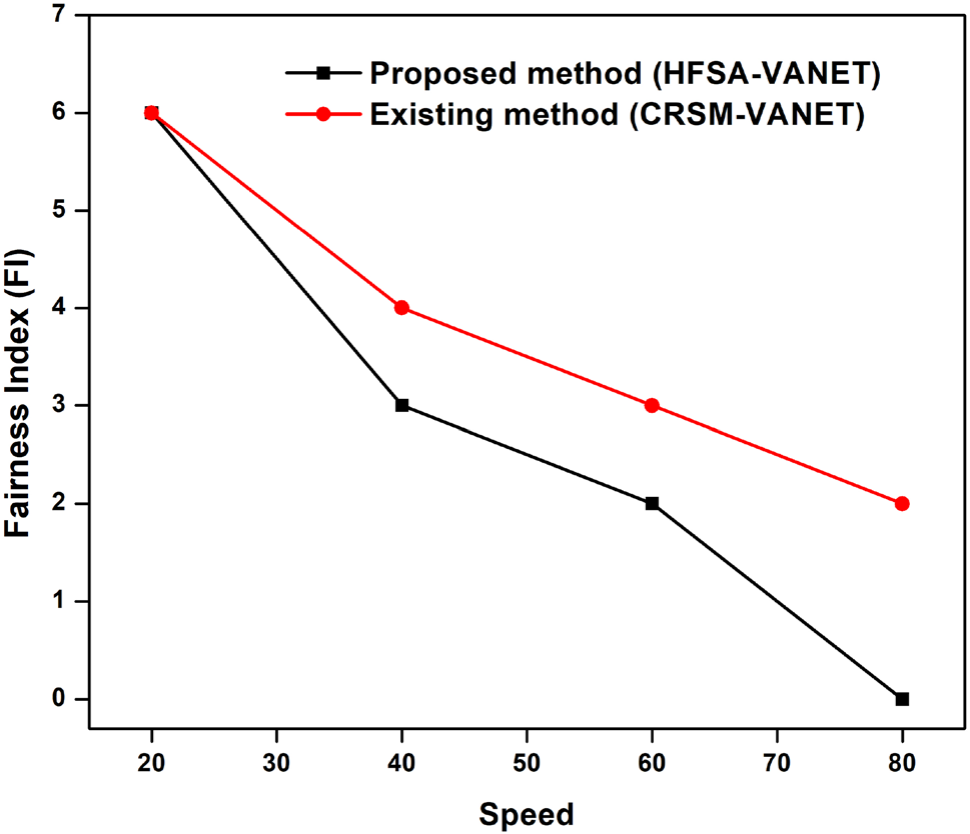
Speed vs fairness index plot for a proposed and existing method.

The speed is compared to the delay shown in Fig. 8, speed vs energy shown in Fig. 9, speed vs drop shown in Fig. 10, speed vs throughput shown in Fig. 11, and speed vs fairness index shown in Fig. 12. The speed is compared to the delay, energy, drop, throughput and fairness index, and the graphical representation is shown below.

In speed 20, the proposed approach achieves 1980 J, 1.873793, 19.954160 in terms of energy consumption, delay value, and drop value. In addition, the new method achieves a Throughput of 150, which is greater than the previous method. The suggested approach has a fairness score of 6.000000, whereas the present method likewise has a 6.000000 number. The suggested technique achieves 1880 J, 390.117000, 18.883762 for energy consumption, delay value, and drop value in speed 40. Furthermore, the new approach achieves a Throughput of 35, which is higher than the existing method. The recommended technique has a fairness value of 3.000000, but the current method has a score of 4.000000. In speed 60, the suggested approach achieves 2220 J, 654.169557, 22.597974 in terms of energy consumption, delay, and drop value. In addition, the proposed strategy yields a Throughput of 22 vs 16 for the present method. The suggested technique has a fairness index of 2.000000, whereas the present method has a fairness index of 3.000000. The recommended method achieves 880 J, 1223.026093, 9.309993 for energy consumption, delay value, and drop value in speed 80. Furthermore, the new technique achieves a Throughput of 8 and the existing technique achieves a throughput of 6. The suggested technique has a fairness score of 0.000000, whereas the current method has one of 2.000000. The Figs. 8, 9, 10, 11 and 12 are Delay, Energy Consumption, Drop, Throughput, Fairness Index are obtained through speed, respectively. The Section 2 covers the results obtained through the MATLAB software.

### Section 2

This section covers the experimental results obtained through MATLAB (VERSION 2020a) for evaluating the performance with the NS2 tool. Moreover, we also include an additional parameter to ensure the network lifetime of the proposed model. Therefore, the performance can be proven as highly effective as the existing technique. Here, the performance of the proposed model is evaluated using various machine learning approaches such as ANN-HFSA-VANET, SVM-HFSA-VANET, NB-HFSA-VANET, and DT-HFSA-VANET. Thus, the proposed model results can be compared and proven as more effective than all other existing techniques.

**Figure 13:**
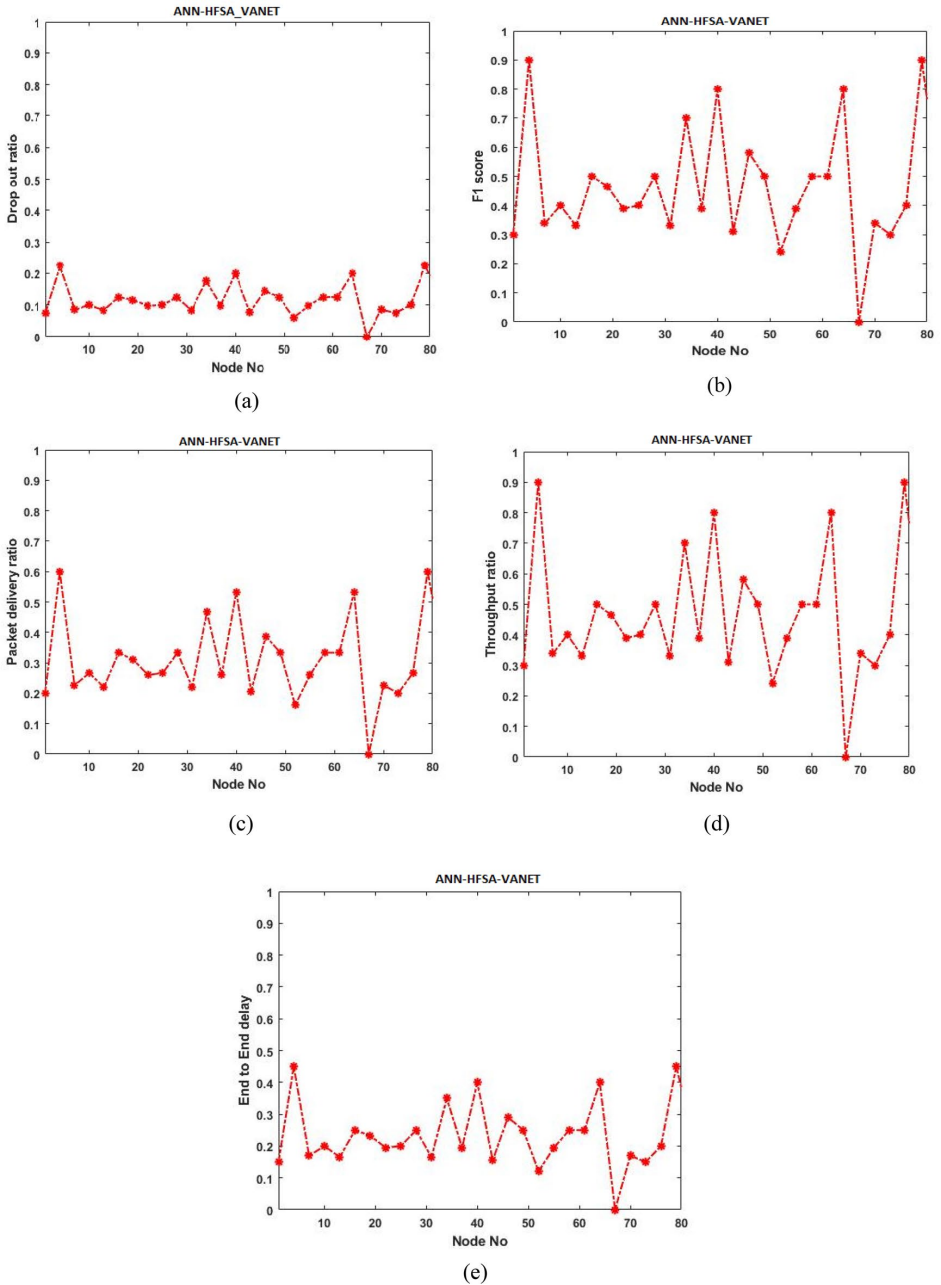
(**a**) Dropout ratio, (**b**) F1 score, (**c**) packet delivery ratio, (**d**) Throughput ratio, (**e**) End to end delay.

**Figure 14:**
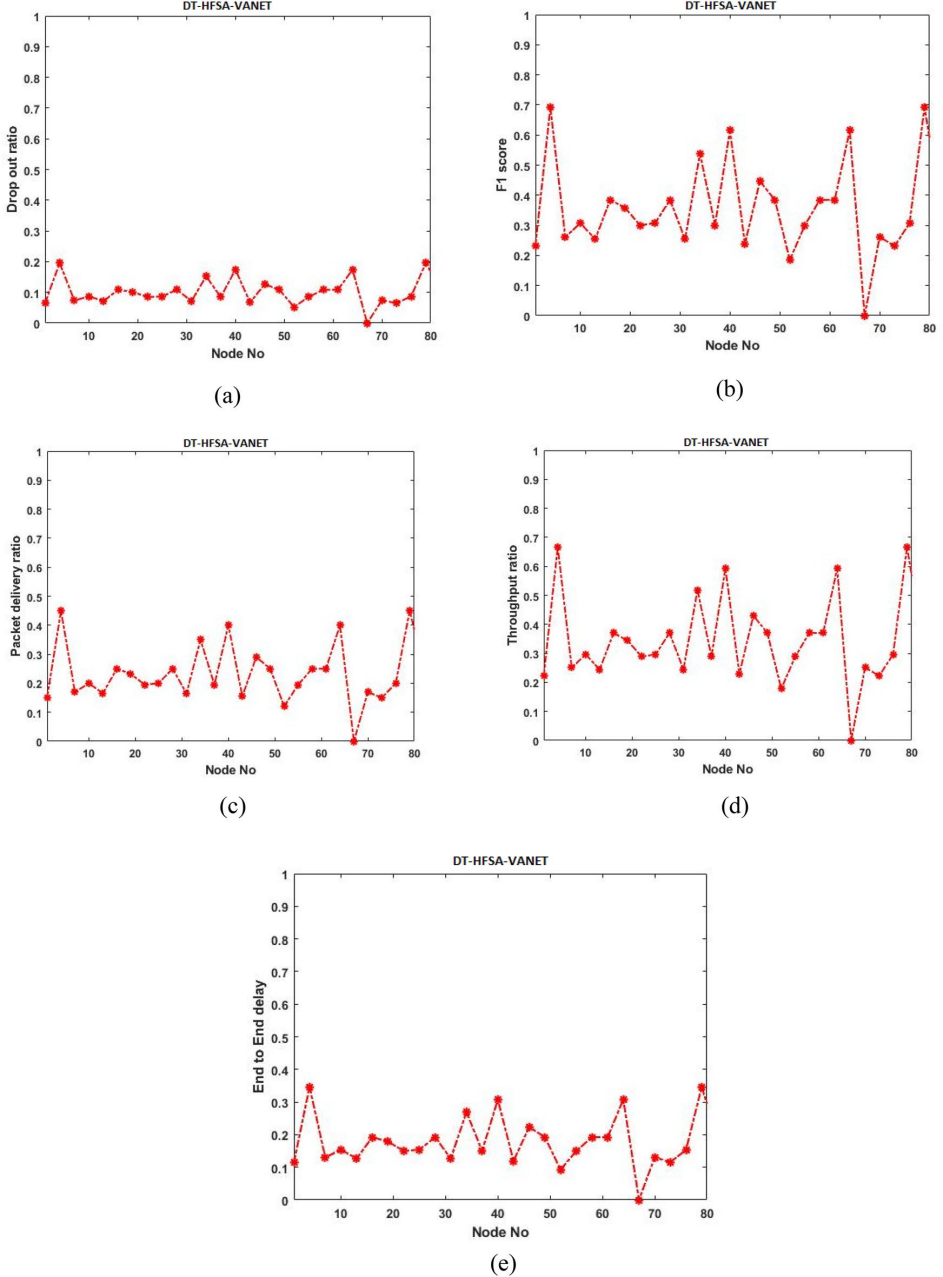
(**a**) Dropout ratio, (**b**) F1 score, (**c**) packet delivery ratio, (**d**) Throughput ratio, (**e**) End to end delay.

**Figure 15:**
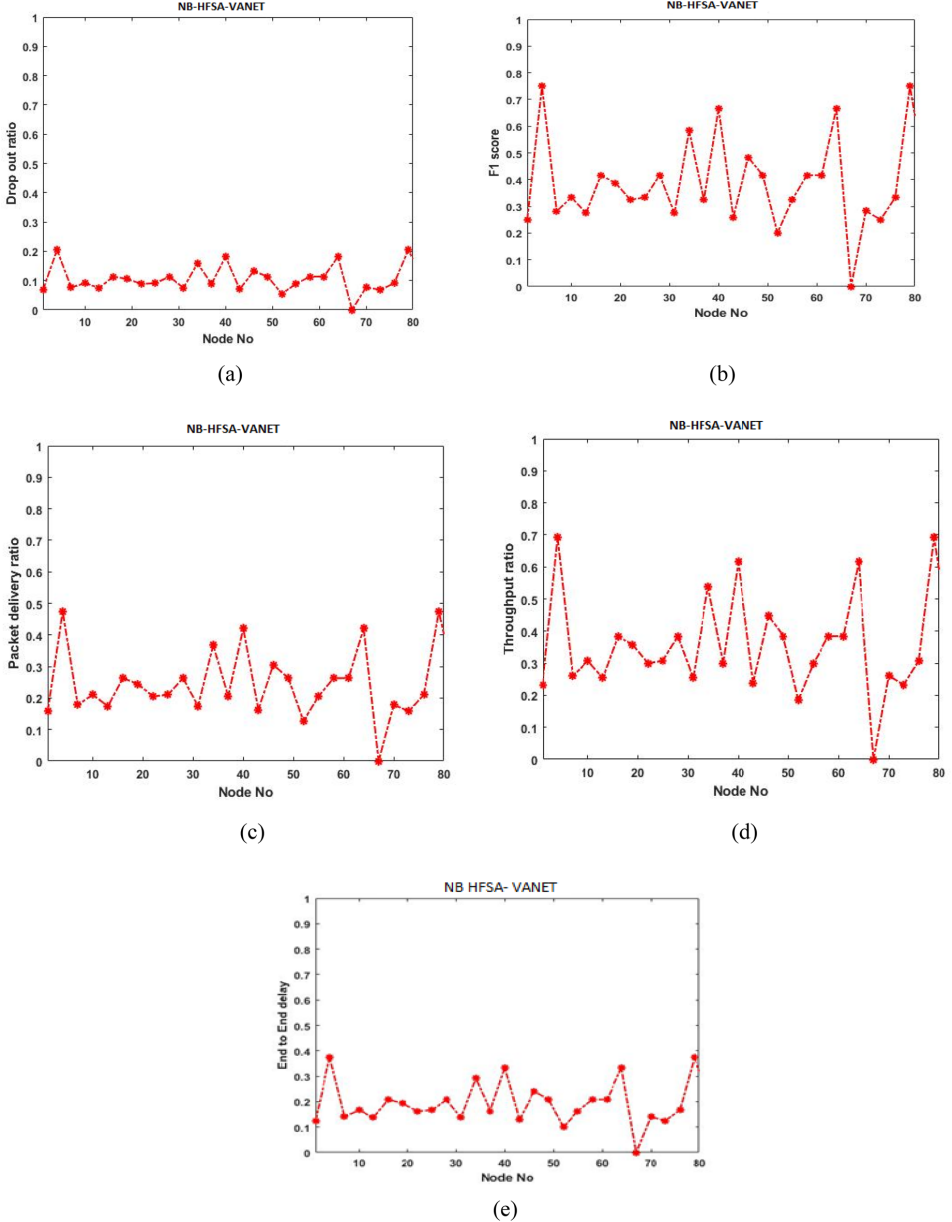
(**a**) Drop out ratio, (**b**) F1 score, (**c**) packet delivery ratio, (**d**) Through put ratio, (**e**) End to end delay.

**Figure 16:**
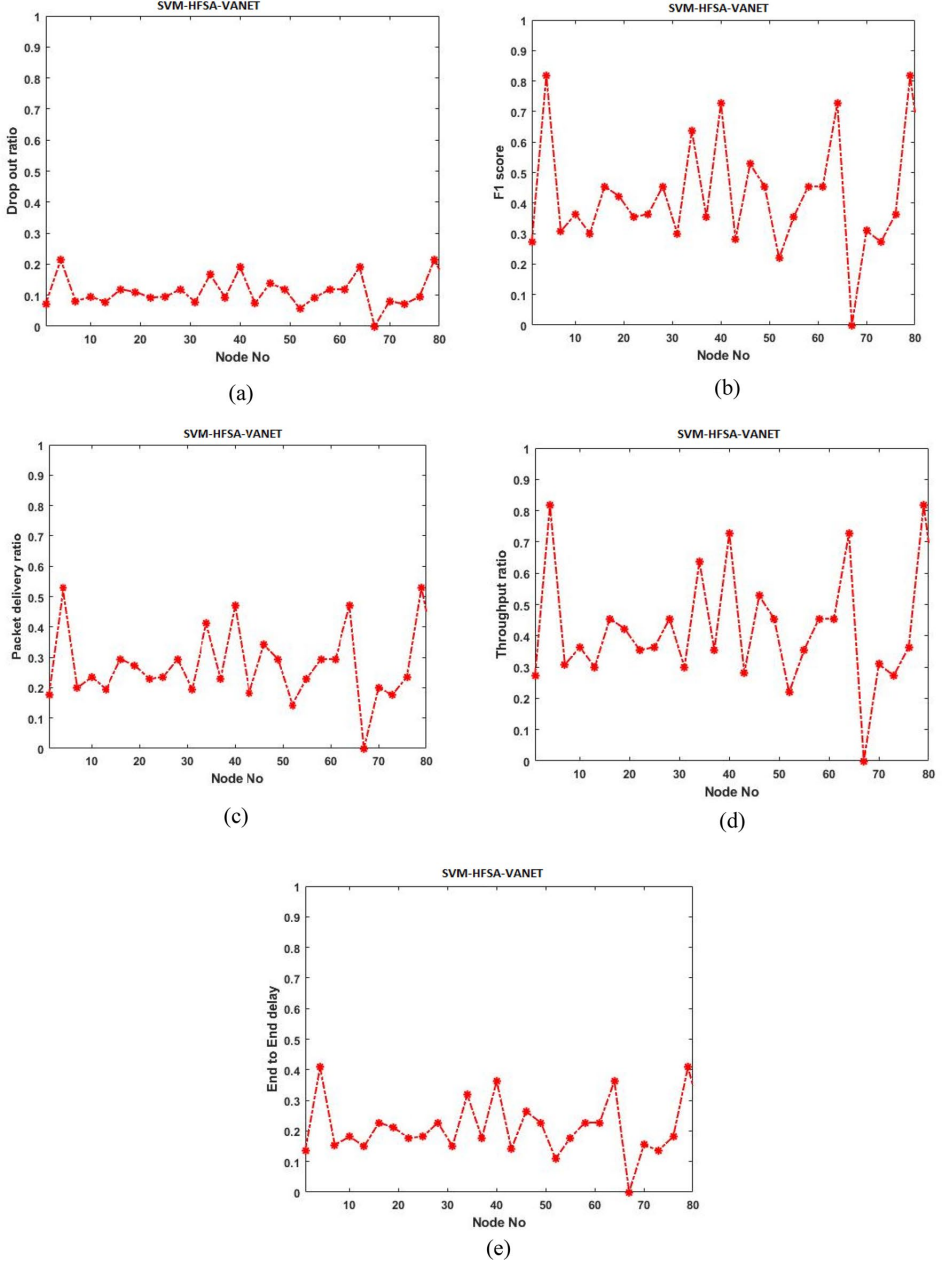
(**a**) Dropout ratio, (**b**) F1 score, (**c**) packet delivery ratio, (**d**) Through put ratio, (**e**) End to end delay.

Initially, the proposed model is evaluated with ANN-HFSA-VANET, SVM-HFSA-VANET, NB-HFSA-VANET, and DT-HFSA-VANET separately. The following Figs. 13, 14, 15 and 16 are showing the graphical results of ANN, SVM, NB, and DT, respectively. On the other hand, to show comparison based on the aggregation of various machine learning techniques that the proposed method is comparing with the single graphical results.

#### Parameter analysis of ANN-HFSA-VANET

This section deals with the different types of parameters of ANN-HFSA-VANET and is analyzed in the graph shown in Fig. 13.

The figure mentioned above 13 illustrates the various performance analysis based on ANN-HFSA-VANET where (a) shows that the proposed technique has obtained minimum dropout, (b) shows maximum F1 score has been obtained by using the proposed technique, (c) illustrates that maximum packet delivery ratio has obtained for the ANN- HFSA-VANET, (d) and (e) show that proposed ANN-HFSA-VANET has generated high throughput and minimum delay, respectively.

#### Parameter analysis of Decision Tree (DT)-HFSA-VANET

This section deals with the different types of parameters of the Decision Tree and analyzed in the graphs shown in Fig. 14.

Figure 14, shows the analysis of parameters of DT-HFSA-VANET. (a) shows the mimumum drop out ratio of the DT-HFSA-VANET, (b) deals with the maximum score for F1 score of DT-HFSA-VANET and its analysis, (c) shows packet delivery ratio of DT-HFSA-VANET and its values plotted, (d) deals with throughput ratio of DT-HFSA-VANET, (e) deals with end to end delay of DT-HFSA-VANET. Standard parameters are analyzed and plotted in a graph and the values increased at the end of each graph of the parameter.

#### Parameter analysis of Navie Baves (NB)-HFSA-VANET

This section deals with the different types of the parameter of Navie Baves and is analyzed in the graph shown in Fig. 15.

In Fig. 15a shows that minimum dropout, (b) shows that maximum F1 score, (c) provides maximum packet delivery ratio, (e) shows that minimum delay, respectively for the proposed NB-HFSA-VANET.

#### Parameter analysis of SVM

This section deals with the different types of a parameter of SVM and is analyzed in the graph shown in Fig. 16.

In Fig. 16a dropout ratio has obtained with minimum ratio, (b) F1 score has obtained with maximum score, (c) packet delivery ratio has obtained with maximum, and (e) shows minimum delay.

### Parameters for analyzing different data types

This section deals with the parameters of different data and their analysis. The values are plotted in a graph.

**Figure 17:**
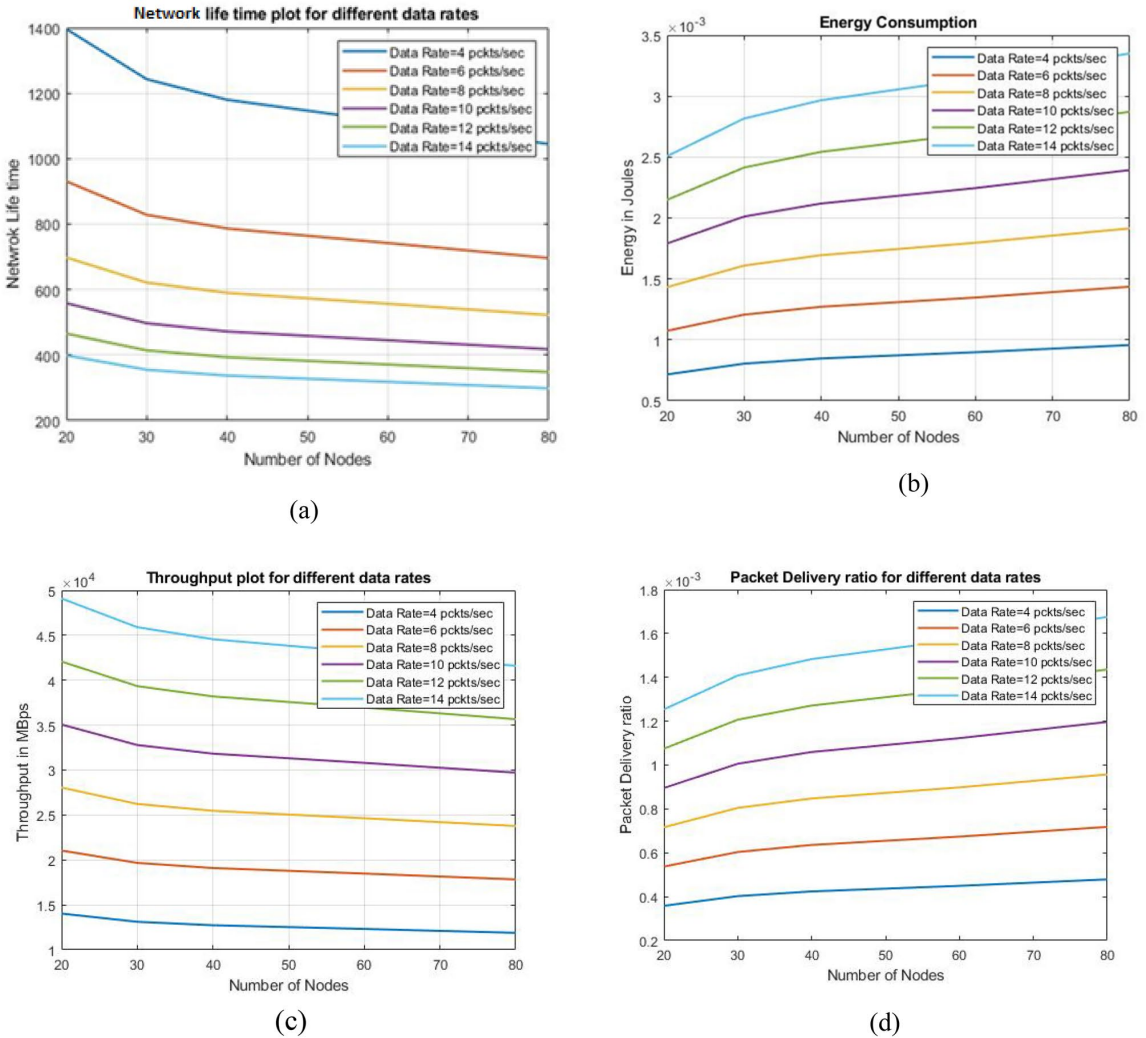
(**a**) Network life time plot, (**b**) Energy consumption, (**c**) Throughput ratio, (**d**) Packet delivery ratio.

From Fig. 17, the parameter analysis value of data types is examined and plotted in a graph where (a) denotes network life time obtained for every second, (b) deals with energy consumption of data packets used per second, (c) deals with through put ratio of data types and their performance, (d) deals with packet delivery ratio of different types of data performance.

## Comparison of algorithms using parameters

This section deals with the comparison of algorithms using different parameters. The values are plotted in graphs. Algorithms such as ANN-HFSA-VANET, SVM-HFSA-VANET, NB-HFSA-VANET, DT-HFSA-VANET are taken into consideration for comparison.

**Figure 18:**
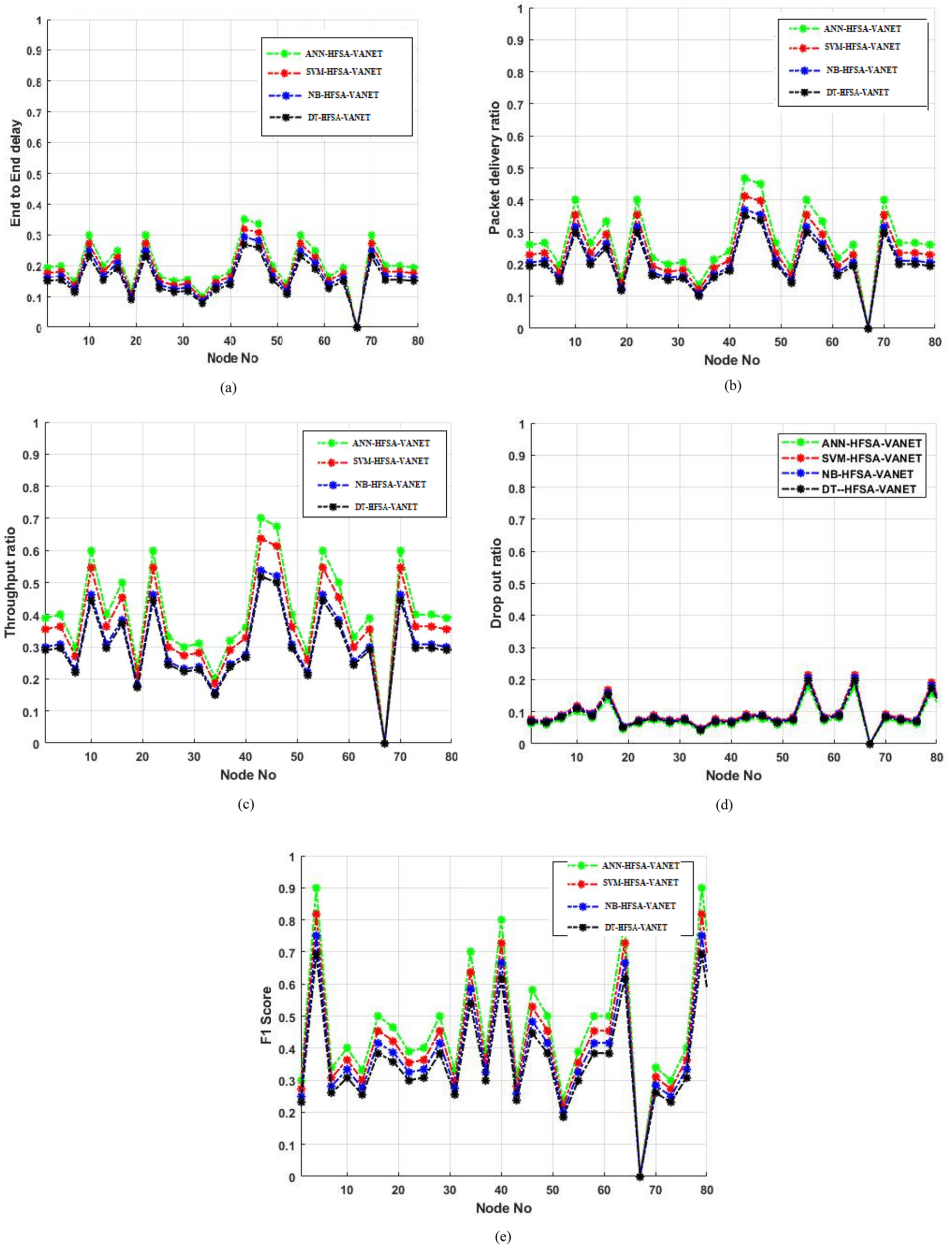
Comparison of algorithms using parameters (**a**) End to End delay, (**b**) Packet delivery ratio, (**c**) Throughput ratio, (**d**) Drop out ratio, (**e**) F1 score.

Figure 18a deals with the end to an end delay parameter of the existing algorithms, (b) dispalys the packet ratio of the existing algorithm and their comparison, (c) deals with throughput ratio and performance of algorithms that exist in it, and (d) describes drop out ratio and the comparison of algorithms and their performance and (e) shows the F1 score of the comparison of the algorithms.

## Conclusion

The proposed ensemble with HFSA-VANET is highly effective and ensuring network stability by periodically monitoring every node inside the network. The HFSA is incorporated with Artificial Fish Swarm Optimization (AFSO) and Seagull Optimization. The seagull optimization technique is utilized for enhancing the performance of the AFSO which is applied for VANET enhancement. Thus, provides highly effective results that are analyzed through an ensemble learning approach that has ANN, SVM, DT and Naïve Bayes. The implementation is done in the platform of NS2 and MATLAB. The HFSA-VANET is implemented without ensemble learning in NS2 and HFSA-VANET with Ensemble Learning in MATLAB for showing comparative results of the proposed algorithm. The HFSA-VANET method shows an overall drop in the delay of 33% and a decrease in the energy consumption of 81% and an increase of 8% in the throughput as compared with the CRSM-VANET method at 80 node. Therefore, the proposed model can be proven as a highly effective technique than the existing techniques.

## Data Availability

The data associated with this work will be made available on request. The datasets generated during and/or analyzed during the current study are available from the corresponding author on reasonable request.
